# Deciphering the crucial roles of transcriptional regulator GadR on gamma-aminobutyric acid production and acid resistance in *Lactobacillus brevis*

**DOI:** 10.1186/s12934-019-1157-2

**Published:** 2019-06-13

**Authors:** Luchan Gong, Cong Ren, Yan Xu

**Affiliations:** Key Laboratory of Industrial Biotechnology of Ministry of Education, State Key Laboratory of Food Science and Technology, School of Biotechnology, Jiangnan University, Wuxi, 214122 China

**Keywords:** *Lactobacillus brevis*, GadR, Acid resistance, γ-Aminobutyric acid

## Abstract

**Background:**

In lactic acid bacteria (LAB), acid stress leads to decreases of cell vitality and fermentation yield. Glutamate decarboxylase (GAD) system is regarded as one of the essential acid-resistance mechanisms in LAB. However, the regulation of GAD system is not well identified in the genus *Lactobacillus*. Although potential transcriptional regulator gene located upstream of GAD system genes was found in several *Lactobacillus* species, such as *Lactobacillus* (*L.*) *brevis*, the contribution of the regulator to acid resistance of the genus *Lactobacillus* has not been experimentally determined.

**Results:**

The potential transcriptional regulator gene *gad*R was disrupted by homologous recombination in *L. brevis* ATCC 367, leading to the decreased expression of *gad*C and *gadB*. The inactivation of GadR completely eliminated γ-aminobutyric acid (GABA) production and decreased the glutamate-dependent acid resistance. Moreover, expression of *gad*C and *gad*B in the presence of glutamate was increased and glutamate also stimulated the expression of *gad*R. In addition, *L. brevis* D17, a strain screened from acidic fermented grains of Chinese liquor production, had much higher expression level of *gad*R than the typical strain *L. brevis* ATCC 367. Under the pH-controlled and mixed-feed fermentation, *L. brevis* D17 achieved a titer of 177.74 g/L and a productivity of 4.94 g/L/h of GABA within 36 h. However, the *L. brevis* ATCC 367 only achieved a titer of 6.44 g/L and 0.18 g/L/h of GABA although the same fermentation control approach was employed.

**Conclusions:**

GadR is a positive transcriptional regulator controlling GABA conversion and acid resistance in *L. brevis*. *L. brevis* strains with hyper-expressing of *gad*R are excellent candidates for GABA production in industrial scale.

**Electronic supplementary material:**

The online version of this article (10.1186/s12934-019-1157-2) contains supplementary material, which is available to authorized users.

## Background

Lactic acid bacteria (LAB) play crucial roles in food processing as generally-regarded-as-safe (GRAS) organisms and health-promoting probiotics [1]. During the fermentation, lactic acid and other acids accumulate in the intracellular and extracellular environment, leading to a huge survival challenge for LAB [2, 3]. Otherwise, acids can cause some detrimental effects, such as denaturing acid-sensitive enzymes, damaging proteins and DNA, and changing the cellular physiology of LAB [4, 5]. Thus, studying the acid resistance mechanisms and protecting LAB survival in the acidic environment are essential.

LAB employ various types of acid resistance mechanisms to counteract the acidic stress, including the F_1_-F_0_-ATPase proton pump, the glutamate decarboxylase (GAD) system, the alkali production pathways, the formation of exopolysaccharides (dextran, reuteran, and levan), and repairing macromolecules [6, 7]. Among these mechanisms, the GAD system is regarded as one of the essential acid resistance mechanisms in LAB [8, 9]. GAD system consists of GAD encoded by *gad*B/A and glutamate/GABA antiporter encoded by *gad*C [10]. GAD catalyzes the decarboxylation of glutamate to produce GABA. Meanwhile, this decarboxylation reaction consumes protons generating proton motive force (PMF) to elevate intracellular pH. The pH elevation can help to reduce viability decline of cells in the acidic environment. In addition, three decarboxylation-antiporter reactions generate one ATP [11]. Therefore, the exertion of the GAD system not only protects cells from damage by acids but also generates energy. Moreover, the byproduct of the decarboxylation reaction, GABA acted as an inhibitory neurotransmitter in human central nervous system has various physiological functions, including antioxidant, hypolipidemic, anti-inflammatory, diuretic and tranquilizer effects [12–15]. Considering the GRAS status of LAB and potentially used as starters for fermented foods with functional properties, GABA-producing LAB has been receiving more and more attention in recent years [16, 17]. Many researchers focus on isolating the GABA hyper-producing strains, optimizing the medium composition and fermentation condition for GABA production, characterizing GAD, and increasing the activity of GAD by genetic modification [16, 18, 19].

Previous studies have shown that GABA-producing strains, including *Lactococcus* (*Lc.*) *lactis*, *L. brevis*, *L. buchneri*, *L. helveticus*, *L. paracasei*, *L. plantarum*, and *Streptococcus* (*S.*) *thermophiles,* are frequently isolated from kimchi, cheese, and *paocai* [12, 17, 20–28]. Among these LAB species, *L. brevis* has been found to be the most frequently isolated species with efficient GABA-producing capability [17, 20, 24]. *L. brevis* contains two GAD encoding genes, *gad*A and *gad*B, sharing approximately 50% protein sequence identity, whereas the GAD activity is mainly contributed by GadB linked to GadC [5]. The *gad*C-*gad*B operon (*gad*CB) including *gadB* encoding glutamate decarboxylase and *gadC* encoding glutamate/GABA antiporter has been reported to be activated by the transcriptional regulator GadR in *Lc. lactis* [10]. According to genomic context analysis, potential transcriptional regulator genes can be found the upstream of the *gad*CB in several *Lactobacillus* species and are usually annotated as *gadR*, such as in *L. brevis* [8, 29]. However, the amino acids sequence identities of these potential transcriptional regulators are extremely low, e.g. only 10% identity can be found between the regulator (annotated as GadR) from *L. brevis* and verified GadR from *Lc. lactis.* Therefore, it still remains unknown whether the annotated *gadR* gene encodes a transcriptional regulator in the genus *Lactobacillus* or not. GAD system is an important acid-resistance system in the genus *Lactobacillus*, however, experimental evidences of the contribution of GadR to acid resistance in these *gad*R-containing species are lacking.

In this study, we investigated the function of GadR in *L. brevis*. The *gad*R deletion strain was constructed to reveal the roles of GadR. We found that the active expression of *gadR* was closely correlated with GABA production and acid resistance in *L. brevis*. Much higher titer and productivity of GABA could be achieved by hyper-expressing GadR strain via fermentation control.

## Results

### Disruption of *gadR* eliminates GABA production

**Fig. 1:**
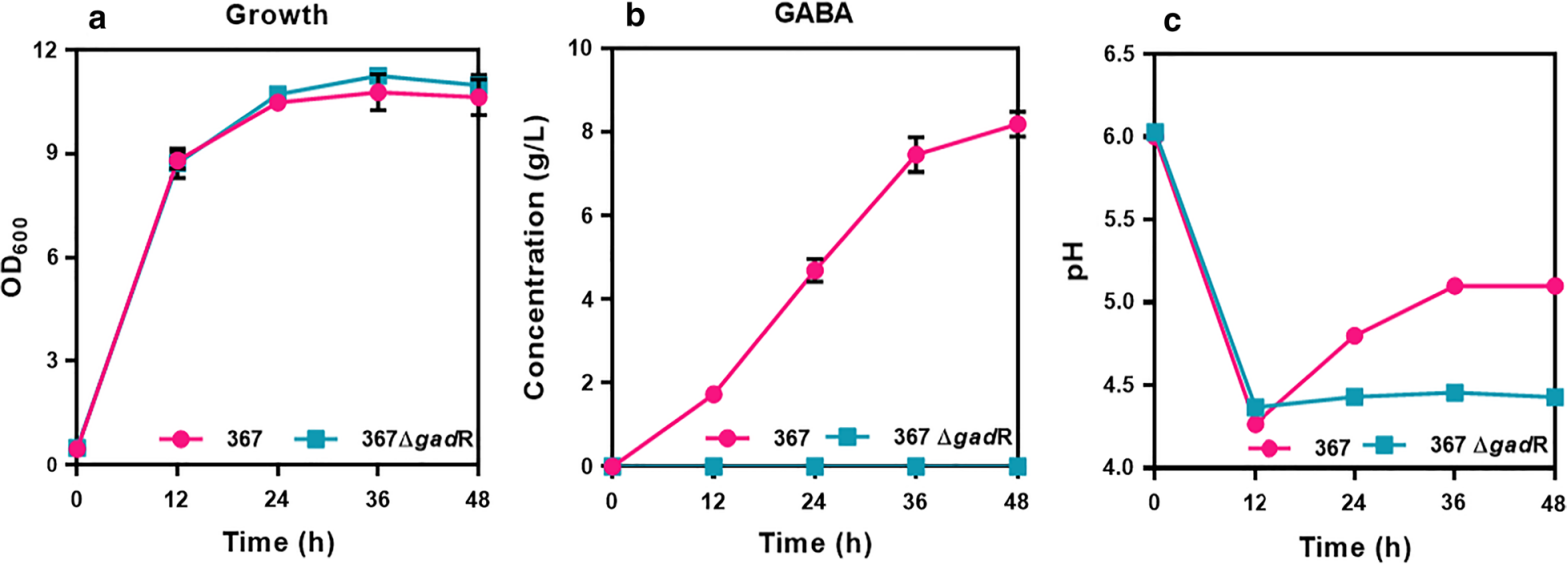
Effect of *gad*R-deletion on *Lactobacillus brevis* ATCC 367. **a**–**c** Fermentation phenotype between strain 367 and strain 367Δ*gad*R with monosodium glutamate (MSG). Δ1 and Δ2, strain 367Δ*gad*R; wt, wild-type strain 367

Based on the genomic context analysis, the gene organization of *gad*R-*gad*C-*gad*B in *L. brevis* genome showed the same organization like that in *Lc. lactis* [8, 10]. However, no experimental evidences were shown to confirm the function of the potential regulator GadR in the genus *Lactobacillus*, e.g. *L. brevis*. To determine the function of GadR, the potential regulator GadR encoding gene was deleted in *L. brevis* ATCC367. A truncated DNA fragment (2 kb) was detected from *gad*R deletion strains (367Δ*gad*R) while an intact DNA fragment (2.5 kb) was detected in the wild-type strain (367) (see Additional file 1). Compared with strain 367, the *gad*R hardly expressed in strain 367Δ*gad*R (see Additional file 1). These results showed that *gad*R was successfully disrupted in strain 367Δ*gad*R. The cell growth was almost similar between strain 367 and strain 367Δ*gad*R (Fig. 1a), indicating that the disruption of *gad*R did not affect the cell growth in *L. brevis*. However, fermentation phenotype for the GABA conversion from glutamate was significantly different between strain 367 and strain 367Δ*gad*R. For strain 367, the titer of GABA reached 8.20 g/L at 48 h, while GABA production capability was completely eliminated in strain 367Δ*gad*R (Fig. 1b). Moreover, the pH of the fermentation broth was gradually elevated from 4.30 to 5.10 for strain 367 due to consuming of protons, whereas pH of the broth remained about 4.30 for strain 367Δ*gad*R (Fig. 1c). These results indicated that the annotated regulator GadR was an activator for GABA conversion from glutamate. We then presumed that GadR could positively regulate the expression of *gad*C and *gad*B for GABA conversion in *L. brevis*.

### The expression of *gad*CB is positively controlled by GadR in a glutamate-dependent manner

**Fig. 2:**
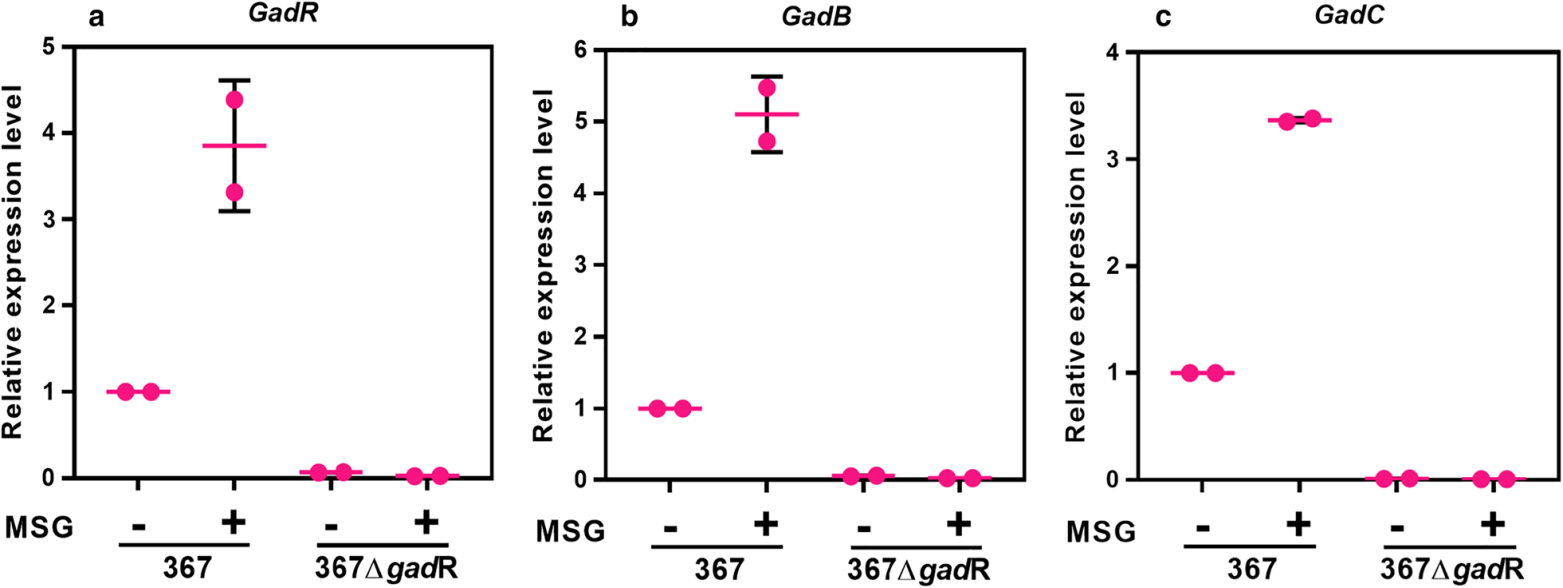
Expression levels of *gad*R, *gad*B and *gad*C between strain 367 and strain 367Δ*gad*R in the presence (+) or absence (−) of MSG

To determine if the *gad*CB operon is regulated by the regulator GadR, the expression levels of *gad*C, *gad*B and *gad*R were examined in strain 367 and strain 367Δ*gad*R. In strain 367Δ*gad*R, no obvious expression of *gad*C and *gad*B was found in the presence or absence of monosodium glutamate (MSG) (Fig. 2), indicating that *gad*R is essential to the expression of the *gad*CB. Although the amino acid sequences of GadR were significantly different in *L. brevis* and *Lc. lactis*, it seems that GadR performed similar regulatory function as a transcriptional regulator for *gad*CB in these two LAB species. The expression of *gad*R was found to be induced by glutamate in a time-dependent manner in strain 367 (Fig. 2a). Meanwhile, the expression levels of *gad*C and *gad*B in the presence of MSG were 3.36-fold and 5.10-fold higher than that in the absence of MSG in strain 367, respectively (Fig. 2b, c). Hence, the expression patterns of *gad*CB in strain 367 and strain 367Δ*gad*R demonstrated that the transcription of *gad*CB was positively regulated by GadR and meanwhile induced by glutamate in a time-dependent manner. GadR positively regulated the transcription of *gad*CB in both *L. brevis* and *Lc. lactis*, however, the expression of *gad*R in *Lc. Lactis* cannot be induced by glutamate [10]. Together, in *L. brevis*, the glutamate-dependent expression of *gad*CB was controlled via transcriptional regulation of its activator GadR and the high expression of *gad*R could elevate the expression levels of *gad*CB.

### Actively expressed GadR is essential to cell survive in acidic environment

**Fig. 3:**
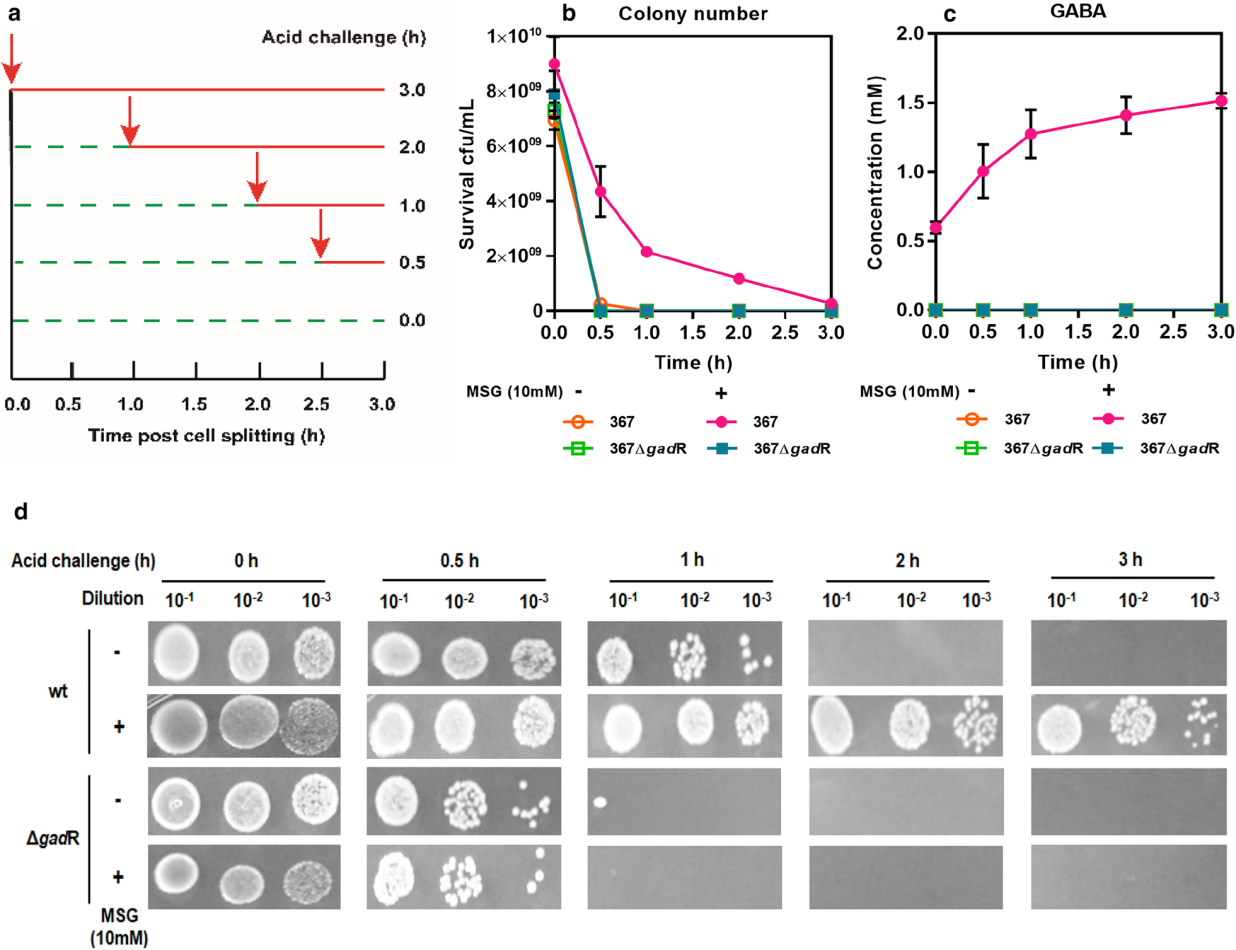
Acid resistance of strain 367 and strain 367Δ*gad*R in buffer (pH 2.5) with 10 mM MSG (+) or without MSG (−). **a** Diagram of acid challenge for strain 367 and strain 367Δ*gad*R. Red arrows, the start point of acid challenge; green dotted line, buffer (pH 7.0); Red line, buffer (pH 2.5). **b** Viable cell number of strain 367 and strain 367Δ*gad*R after acid challenge. **c** GABA production of strain 367 and strain 367Δ*gad*R after acid challenge. **d** Survival of strain 367 and strain 367Δ*gad*R after acid challenge. The images represent serial dilutions of the cultures in tenfold steps

GAD system (GadC and GadB) is regarded as a primary acid-resistance mechanism in LAB [8, 9]. To examine the contribution of GadR to acid resistance, acid challenge assay was performed (Fig. 3a). The colony number of strain 367 was significantly higher than that of strain 367Δ*gad*R in the presence of MSG (Fig. 3b), indicating that strain 367Δ*gad*R was more sensitive to acid challenge than strain 367. In addition, there was a good correlation between cell survival and GABA production in *L. brevis*. GABA production was increased in a time-dependent manner in strain 367 (Fig. 3c). However, both strain 367 and strain 367Δ*gad*R were sensitive to acid challenge in the absence of MSG (Fig. 3b). Given that low concentration (10 mM) of MSG was used in the acid challenge assay, we speculated that trace glutamate in natural habitats could effectively protect *L. brevis* cells from acid stress. In addition, cell survival under acid challenge also demonstrated that the actively expressed GadR was vital to the acid resistance in *L. brevis* (Fig. 3d). These results of acid challenge assays indicated that glutamate stimulated the expression of *gad*R and the actively expressed GadR was essential to cell survival under acid challenge. Considering that the transcription of GAD system was positively controlled by GadR, the glutamate-dependent acid resistance in *L. brevis* was mediated via GadR regulation. Furthermore, *gad*R could be used as a novel genetic engineering target to improve the capability of acid resistance in *L. brevis*.

### GadR is hyper-expressed in the strain isolated from acidic habit

Based on the above results, *L. brevis* GadR was a transcriptional regulator contributing to acid resistance in the presence of glutamate by activating the expression of *gadCB*, the operon encoding GAD and antiporter for GABA conversion from glutamate. Considering that the capability of GABA production was correlated with acid resistance, the acidic fermented grains of the traditional Chinese liquor production would be ideal resources to screen LAB strains with high GABA-producing capability. During the fermentation process, the concentration of free glutamate was 500 to 2500 mg/kg, and the pH of the fermented grains quickly decreased to 3.5 and remained constant for 40–60 days [30]. Due to the correlation between acid resistance and GABA production, the GABA-producing capability was used as the screening standard. One hundred forty strains were picked up and 66 strains (47.1%) produced GABA from 0.1–6.56 g/L (data not shown). Among these GABA-producing strains, 19 strains (28.8%) produced GABA more than 1 g/L (see Additional file 2). Although *L. brevis* was found to be the most abundant GABA-producing species, its GABA-producing capability was diverse (see Additional file 2). In particular, *L. brevis* D17 (D17) strain produced GABA at the highest titer of 6.56 g/L among these GABA-producing LAB strains. Previous studies have indicated that acidic habitats such as kimchi, *paocai*, cheese, yogurt, and fermented seafoods are preferred sources for screening hyper GABA-producing strains (see Additional file 3). However, the acidic fermented grains of Chinese liquor production were often ignored for screening high GABA-producing strains. To our knowledge, this is the first study to evaluate GABA-producing LAB strains obtained from the acidic fermented grains of Chinese liquor production.

**Fig. 4:**
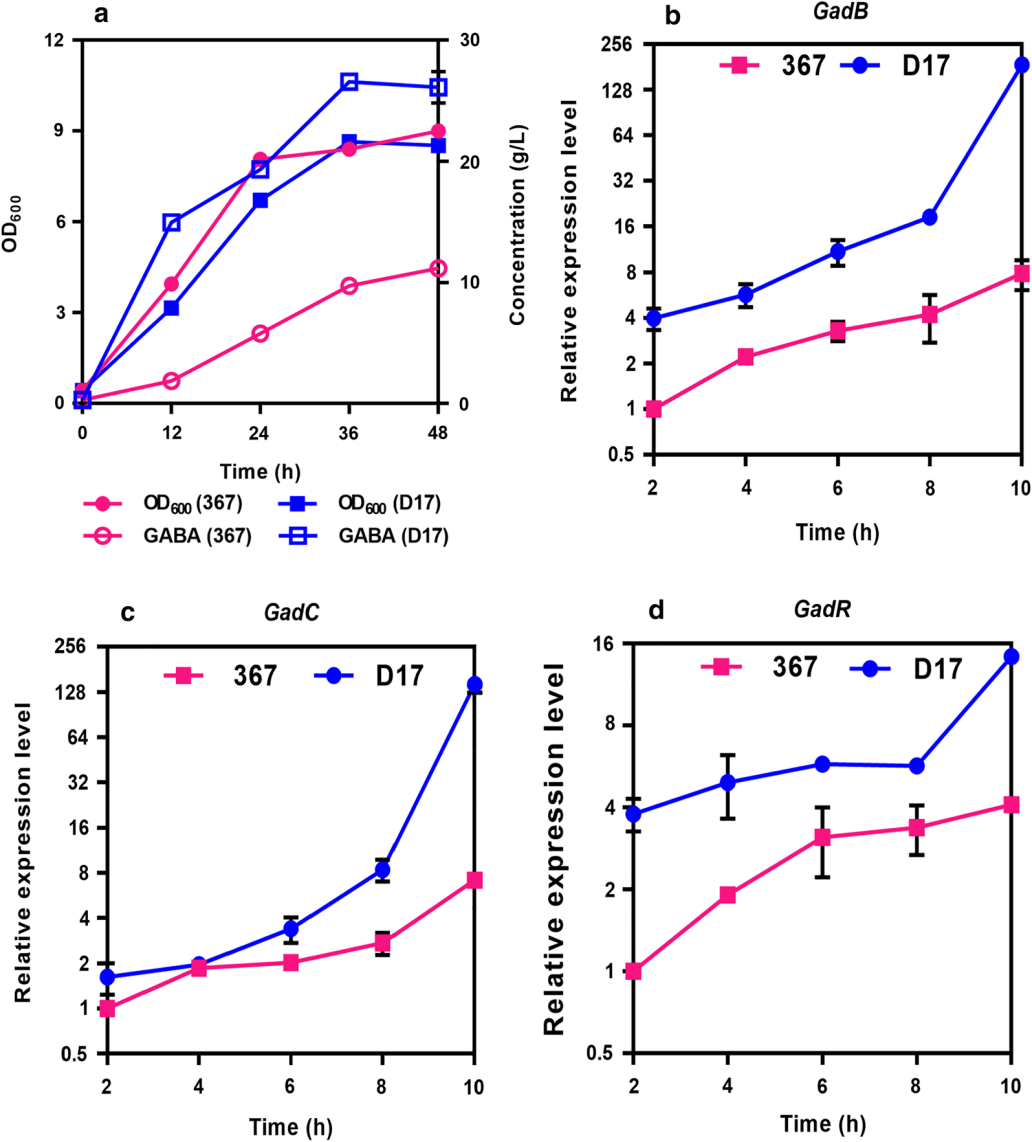
Effect of the *gadR* transcription on GABA production in strain 367 and strain D17. **a** Cell growth and GABA production in strain 367 and strain D17. **b**–**d** Expression levels of *gad*B, *gad*C and *gad*R in strain 367 and strain D17. Strain D17 was isolated from the acidic habit

We then evaluated the capability of GABA production for the strain D17 isolated from acidic fermented grains of Chinese liquor production. The cell growth between strain 367 and strain D17 had no significant difference (Fig. 4a). However, the GABA titer of strain D17 reached 26.1 g/L within a fermentation period of 48 h, 2.3-fold higher than that of strain 367 (11.17 g/L) (Fig. 4a). To examine why strain D17 produced GABA more efficiently, we then compared the amino acid sequences as well as the expression levels of *gad*CB and *gad*R between strain 367 and strain D17. The amino acid sequences of GadCB and GadR were exactly identical (data not shown), suggesting that the different GABA-producing capabilities between strain 367 and strain D17 were not attributed to the primary structures of glutamate decarboxylase and glutamate/GABA antiporter.

We then determined the expression levels of *gad*CB and *gad*R in strain 367 and strain D17. The expression levels of *gad*CB and *gad*R in strain D17 were significantly higher than that in strain 367 (Fig. 4b–d). The *gad*CB and *gad*R were markedly up-regulated in a time-dependent manner both in strain 367 and strain D17. For the expression of *gadB*, we found a 7.8-fold increase in strain 367 and a 47.2-fold increase in strain D17 from 2 h to 10 h. For *gadC*, a 7.1-fold increase in strain 367 and an 89.1-fold increase in strain D17 were found. For *gad*R, a 4.1-fold increase in strain 367 and a 3.8-fold increase in strain D17 were found. Therefore, the time-dependent induction of *gad*CB in strain D17 was much higher than that in strain 367. The expression levels of *gad*CB gradually increased with the increasing expression of *gad*R in a time-dependent manner. We also noted that the expression level of *gad*R in strain D17 was always higher than that in strain 367. GadR was a positive transcriptional regulator controlling the transcription of *gadCB*, we speculated that the hyper expression of *gad*R in strain D17 could be one of the reasons contributing to higher GABA-producing capability. Indeed, when *gad*R in strain D17 was disrupted, the resulted mutant (D17Δ*gad*R) completely lost the GABA-producing capability (see Additional file 4). Therefore, *L. brevis* GadR was an activator for *gad*CB expression, and the expression level of *gad*R could be vital to achieve higher GABA-producing capability.

### GABA production is greatly elevated in the hyper-expressing GadR strain via fermentation control

**Fig. 5:**
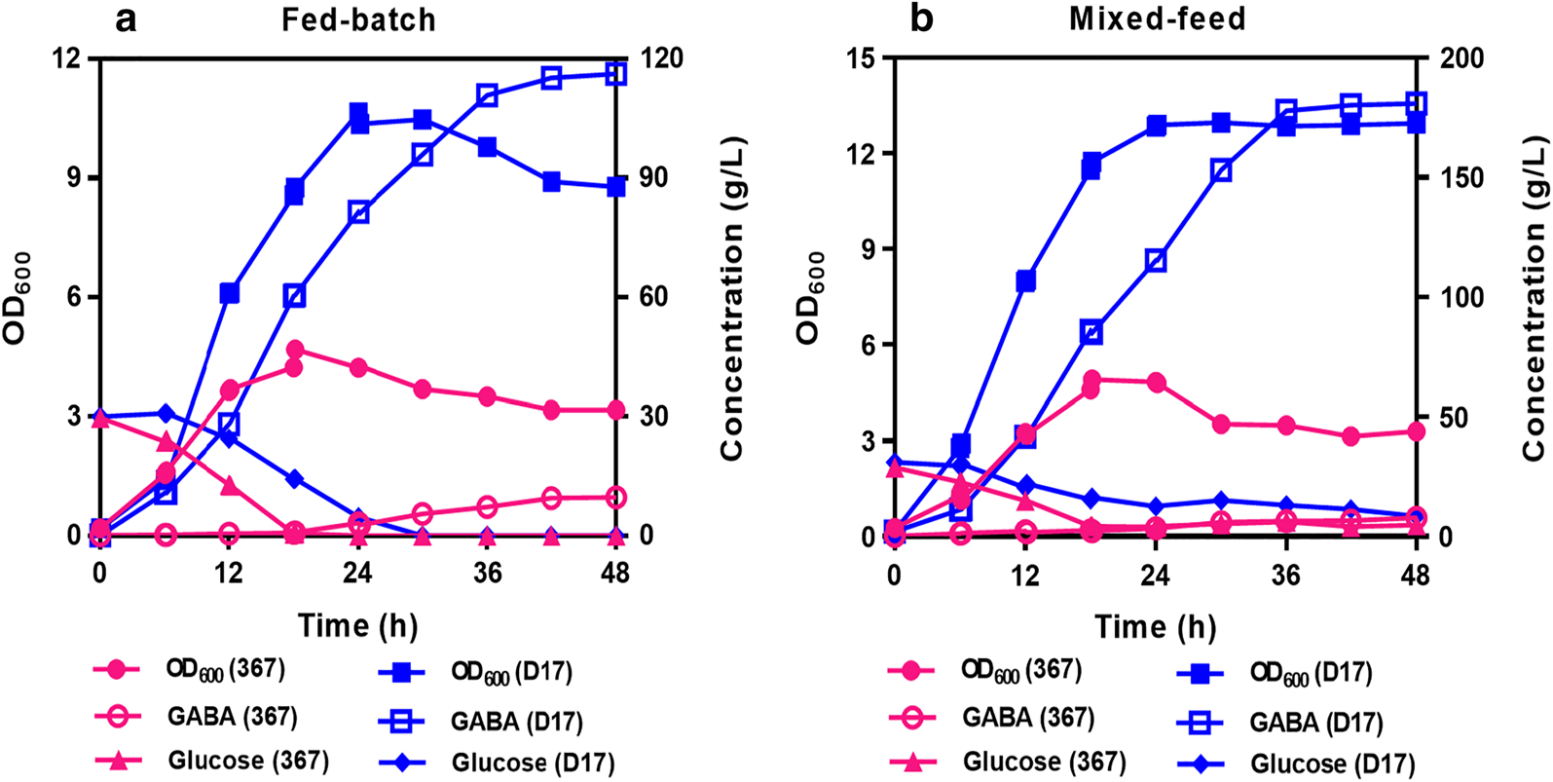
Growth, GABA and carbon source profiles in **a** fed-batch fermentation and **b** mixed-feed fermentation between strain 367 and strain D17. MSG was separately added at 6 h, 12 h, 18 h and 24 h. For mixed-feed fermentation, solution of glucose (300 g/L) was continuously added at a rate of 5 mL/h from 12 h to 36 h

Based on the above data and analysis, actively expressed *gad*R was essential to GABA conversion from glutamate. However, other factors also affect GABA conservation from glutamate, especially the pH, because the decarboxylation of glutamate to GABA requires the same molar amount of proton [31]. The decarboxylation reaction helps increase the alkalinity of cytoplasm and maintain a neutral cytoplasmic pH [31]. The hyper-expression of GAD system is not sufficient to ensure hyper-conversion rate of GABA if H^+^ is limited. It has been reported that the optimum pH of LAB GAD was between 4.0 and 5.0 [32]. A pH of 5.0 controlled by using H_2_SO_4_ was employed in this study [33]. We found that the biomass of strain D17 rapidly increased in fed-batch fermentation (Fig. 5a). Accordingly, in strain D17, GABA rapidly increased within the first 36 h and then have a moderate increase after 36 h (Fig. 5a). Finally, the titer of GABA reached 116.16 g/L and the productivity reached 2.42 g/L/h for strain D17 within a 48 h’s fermentation (Fig. 5a), which were 12-fold higher than that for strain 367. Only a titer of 9.65 g/L for GABA production was achieved for strain 367 although the same pH control and fed-batch approach was used. Therefore, the GABA titer of strain D17 could be greatly improved with pH control.

We also found that the productivity of GABA gradually decreased with the consumption of glucose in *L. brevis* D17 (Fig. 5a). Glucose, a preferred carbon source for most LAB, plays important roles in maintaining cell viability. We then investigated whether mixed-feed fermentation by controlling the carbon source (glucose) availability could be used to further elevate the GABA production. By feeding sufficient glucose in mixed-feed fermentation, the cell density (OD_600_) was up to 12.83 (Fig. 5b), a 19.3% increase compared with that in fed-batch fermentation. The titer of GABA was up to 177.74 g/L at 36 h (Fig. 5b), which was 53.0% higher than that in fed-batch fermentation (Fig. 5a). The productivity of GABA reached 4.94 g/L/h (Fig. 5b), which was 104% higher than that in fed-batch fermentation (2.42 g/L/h, Fig. 5a). Although the same fermentation strategy by feeding sufficient glucose was used to maintain cell viability for strain 367, the *gad*R was insufficiently expressed to elevate the GABA production under the pH-controlled condition (Fig. 5b).

**Fig. 6:**
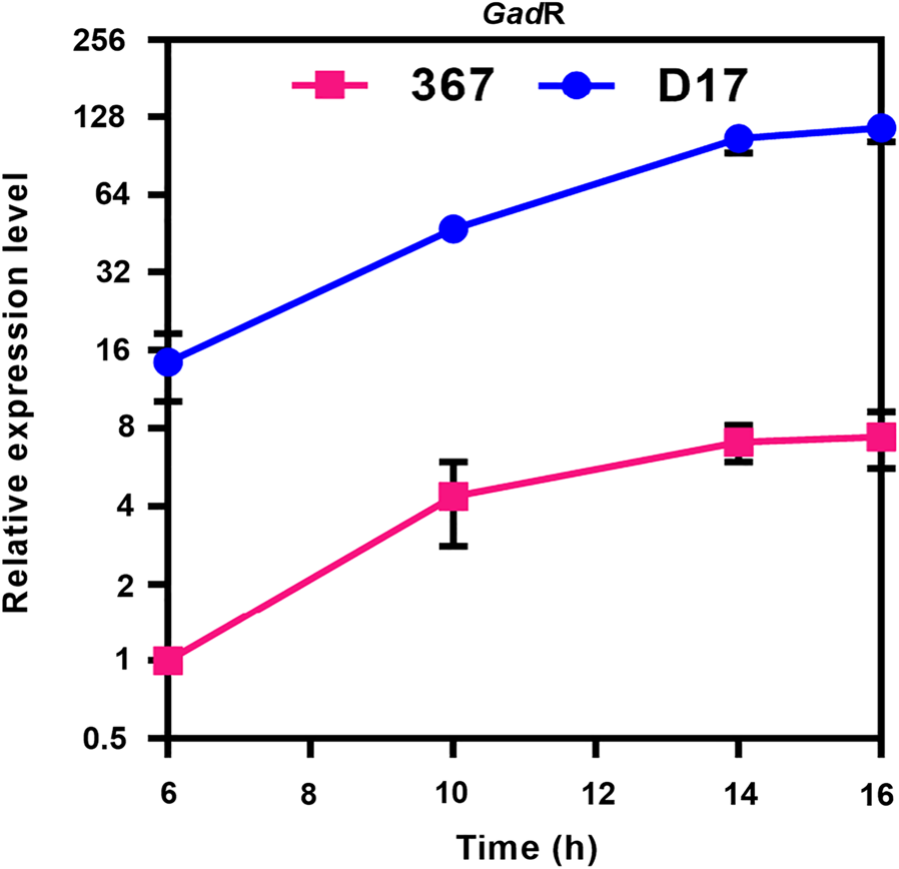
Expression levels of *gad*R in strain 367 and strain D17 cultured in GYP broth with pH control. The pH was controlled at 5.0 by addition of H_2_SO_4_ from the beginning of inoculation

We then determined the expression levels of *gad*R post inoculation and found that *gad*R maintained much higher expression levels in strain D17 than that in strain 367 in fed-batch fermentation (Fig. 6). Compared with the expression level of *gad*R under the pH-uncontrolled condition (Fig. 4d), the expression level of *gadR* under the pH-controlled condition in strain D17 was much higher than that in strain 367 (Fig. 6). The fermentation and transcriptional analysis suggested that *L. brevis* strains with hyper-expressing of *gad*R could be excellent candidates for GABA production in industrial scale.

## Discussion

Besides pH and continuous fed-batch controls, other approaches, e.g. supplement of pyridoxal-5′-phosphate (a factor for GAD) and two-stage pH/temperature control, have been explored to improve GABA production [5, 33]. Our study suggested that high titer and high productivity for GABA production could be obtained via controlling pH and feeding carbon source when LAB strain with hyper-expression of *gad*R was used.

In this study, we found that GadR in *L. brevis* was a transcriptional regulator activating the transcription of *gad*CB. In *L. brevis* species, fourteen strains have complete genome sequences from NCBI. All strains (100%) contain the transcriptional regulator GadR. This suggests that the regulation and the function of GadR could be universal in *L. brevis*. We also analyzed other LAB genomes derived from NCBI database. Fourteen species of LAB have the GAD encoding genes usually annotated as *gad*B and the glutamate/GABA antiporter encoding genes annotated as *gad*C, while among which, nine species (64%) contain the *gad*R-*gad*C-*gad*B genome context organization, especially in the genus *Lactobacillus* (see Additional file 5). Interesting, the sequence identities of these GadR proteins were diverse. For instance, only 6.3% sequence identity was found between *Lc. lactis* and *L. brevis* (see Additional file 5), however, these two GadR regulators showed positive transcriptional regulation on the GAD system. Thus, these annotated *gadR* genes could be involved in acid resistance by controlling the transcription of GAD system in other *gadR*-containing LAB species (see Additional file 5).

In addition, metabolic engineering has been widely used to improve GABA production in *L. brevis*. The *gad*C-*gad*B have been overexpressed in several species to raise GABA conversion from glutamate. For example, overexpression of *gad*CB in *L. brevis* CGMCC 1306 yielded a titer of 105 g/L GABA with pH and temperature controls [5]. *L. brevis* NRA6, an F_0_F_1_-ATPase deficient strain, could only produce GABA at a concentration of 43.65 g/L by overexpressing *gad*B, which is 1.22-fold higher than that obtained by the wild-type strain in the same condition [34]. Meanwhile, a previous study demonstrated that GABA production in *Corynebacterium glutamicum* ATCC 13032 by overexpressing *gad*R-*gad*C-*gad*B was 1.72-fold higher than that by only overexpressing *gad*C-*gad*B, indicating that *gad*R even played a vital role on the ectopic expression of *gadC*-*gadB* [35]. Thus, the *gad*R could be another potential genetic engineering target to elevate GABA production as well as acid resistance in *gad*R-containing LAB species.

## Conclusions

In this study, we determined the contribution of GadR to GABA conversion and acid resistance in *L. brevis*. GadR positively regulates the transcription of *gad*CB in a glutamate-dependent manner. GadR is essential to achieve glutamate-dependent acid resistance in *L. brevis*. This study suggests that high titer and high productivity for GABA production can be achieved via fermentation control when LAB strain has hyper-expression of *gad*R. Therefore, the acidic fermented grains of Chinese liquor production can be used as ideal sources for screening GABA-producing strains and acid resistant strains.

## Methods

### Bacterial strains, media and growth conditions

Strains and plasmids used in this study were listed in Table 1. *Escherichia coli* strains were grown aerobically in Luria-Bertani (LB) medium at 37 °C and 200 rpm. *Lactobacillus brevis* strains were grown aerobically in GYP or MRS medium at 37 °C and 200 rpm. Appropriate concentrations of erythromycin (200 μg/mL for *E. coli* and 1–4 μg/mL for *L. brevis*) were used for the selection of transformants when needed. LB medium contained 10 g/L tryptone, 5 g/L yeast extract and 10 g/L NaCl. The basic MRS medium contained 20 g/L glucose, 10 g/L tryptone, 5 g/L beef extract, 4 g/L yeast extract, 5 g/L sodium acetate anhydrous, 2 g/L ammonium citrate, 2 g/L KH_2_PO_4_, 0.2 g/L MgSO_4_, 0.05 g/L MnSO_4_·H_2_O, 1 g/L Tween-80 and adjusted pH to 6.2 with acetate. GYP medium per liter was composed of 10 g glucose, 10 g yeast extract, 5 g tryptone, 2 g sodium acetate anhydrous, 0.2 g MgSO_4_, 0.1 g MnSO_4_·H_2_O, 0.1 g FeSO_4_·7H_2_O and 0.1 g NaCl. The carbon sources in the MRS and GYP media were separately sterilized to avoid Maillard reactions. Solid medium was prepared by supplementing 2.0% agar (W/V) to the appropriate liquid medium.

**Table 1:** Strains and plasmids used in this study

Strains/plasmids	Characteristics	Sources/references
Strains		
*L. brevis* ATCC 367	Wild-type strain	ATCC
*L. brevis* D17	Wild-type strain	This study
*L. brevis* ATCC 367Δ*gad*R	Derivative of *L. brevis* ATCC 367 with *gad*R deletion	This study
*L. brevis* D17Δ*gad*R	Derivative of *L. brevis* D17 with *gad*R deletion	This study
*E. coli* Top10	Recipient for cloning experiments	Invitrogen
Plasmids		
pGID023	Integration vector, Em^r^ (erythromycin resistance gene)	[38]
pGID023-AB	pGID023 carrying 2 kb DNA fragments derived from upstream and downstream region of the *gad*R gene	This study

### Acid challenge assay and survival of strains

To evaluate acid resistance, *L. brevis* strains were grown in GYP with 50 mM MSG. Early stationary phase cells (10–12 h) were washed by 50 mM potassium phosphate buffer (pH 7.0) and centrifuged at 4 °C, 6000 *g* for 10 min. The obtained cells were suspended in potassium phosphate buffer (pH 7.0) to OD_600_ of 1.0 and incubated at 37 °C [36]. Potassium phosphate buffer (pH 2.5, HCl was used to adjust pH) was used as the acid challenge buffer in acid challenge assay. Ninefold potassium phosphate buffer (pH 2.5) was separately added into onefold cell suspension which has been incubated in potassium phosphate buffer (pH 7.0) for 0 h, 1 h, 2 h and 2.5 h, 3 h at 37 °C. Then these samples would be incubated until the total incubation period of 3 h at 37 °C. The above treating strategy was illustrated in Fig. 3a [37]. MSG (10 mM) was used when necessary. To determine the cell survival, all samples were immediately serially diluted in PBS buffer prior to spread on GYP agar plates followed by culturing for 24 h at 37 °C, and the colonies were counted. In addition, 3 μL of dilutions was separately dripped on GYP agar plates followed by culturing at 37 °C for 24 h, and the plates were photographed [5, 37]. The PBS buffer contained 137 mM NaCl, 2.7 mM KCl, 10 mM Na_2_HPO_4_, and 2 mM KH_2_PO_4_.

### Quantitative reverse transcription PCR

The relative expression levels of *gad*CB and *gad*R mRNA in different stages were examined by quantitative reverse transcription PCR (qRT-PCR). *L. brevis* cells were grown in GYP medium without or with 50 mM MSG at 37 °C and 200 rpm. Cells at different stages were collected by centrifugation at 4 °C, 6000 *g* for 5 min and frozen immediately using liquid nitrogen, and then the cell precipitates were ground into powder. Total RNA was isolated using RNAiso Plus (Takara, Dalian, China) according to the manufacturer’s instructions. The concentration and purity of total RNA were determined by NANODrop 8000 (Thermo, USA). The integrity was verified by agarose gel electrophoresis. cDNA was synthesized with an equal amount of total RNA (0.5 μg) according to the instructions of the PrimeScriptTM RT reagent Kit (Takara, Dalian, China). The gDNA Eraser reagent in the kit was used to digest contaminant DNA in the total RNA prior to reverse transcription. Specific primers for quantitative PCR were designed and listed in Table 2 (qF-16S/qF-16S, qF-*gad*R/qF-*gad*R, qF-*gad*C/qF-*gad*C and qF-*gad*B/qF-*gad*B). The 16S rRNA gene was used as a reference. The quantitative PCRs were performed on the StepOne Plus Real-Time PCR System (Thermo Fisher, USA) following the manufacturer’s protocol. Quantification of expression levels of *gad*R and *gad*CB was conducted using the 2^−ΔΔCt^ method [39].

**Table 2:** Primers used in this study

Primers	Sequence (5′–3′)
F-A-up-*Bam*HI	AGCGCGGATCCACTGGCCATTAAATTAGCCCAAGCCTTTA
R-A-up	CGGCCGCTGTTGCTGCCGTAATTCTTTTTCTTTCACCACTAC
F-B-down	GAAAAAGAATTACGGCAGCAACAGCGGCCGCTTTAAGATATTGATGAG
R-B-down-*Hin*dIII	CCCAAGCTTCCAAGCAACATAGACCGGTGCTTCGT
F-367-0076	CACGCCGACTGAGGTCCTGATTA
R-367-0078	GCTGCCACTTCATCCACATTGACA
F-GadR	GCAGCGGCTAACCGATGATGA
R-GadR	CGGACATGCCTGCTTCAGACTC
qF-16S	TGAGTGCTAAGTGTTGGAGG
qR-16S	ACATCTCACGACACGAGCTG
qF-*gad*R	CGATTCCCATGCTTATTC
qR-*gad*R	TTGCGGAAATGTAACTGC
qF-*gad*C	TCTTAGTGGGATTTGTTCCG
qR-*gad*C	AGCTTTTCGACAAAGACCAC
qF-*gad*B	AGGCTAATCAAAACCTGCG
qR-*gad*B	AACTATGTAGTAGCGCCAAG

### Electroporation

Competent cells of *L. brevis* were prepared as the following. One milliliter overnight cells culture were inoculated into 100 mL MRS medium supplemented with 10 g/L glycine followed by culturing at 200 rpm, 37 °C until OD_600_ reached 0.8. The cells were obtained by centrifugation at room temperature, 4000 *g* for 10 min. Then 100 mL ice-cold buffer (326 g/L sucrose, 0.71 g/L MgCl_4_·6H_2_O) was used the cells to wash 2 times. The cell precipitates were collected by centrifugation at 4 °C, 4000 *g* for 10 min and then suspended in 1 mL ice-cold buffer. For electroporation, 50 μL of the fresh competent cells were mixed with 1 μg plasmid to chill for 5 min on ice. The mixture then was transferred to a pre-chilled electroporation cuvette (0.2 cm, BioRad), and electroporated at 2.5 kV with about 5 ms pulse. After electroporation, 2 mL MRS medium containing 0.3 M sucrose was immediately added to the cuvette. The cell suspension was transferred to a 5 mL sterile tube followed by incubating for 3 h at 37 °C and then spread on MRS agar plates supplemented with 4 μg/mL erythromycin.

### Construction of *L. brevis* gene deletion mutant

Strains and plasmids used in this study were listed in Table 1. Marker-less deletion of *gad*R in *L. brevis* was performed by homologous double crossover according to the previous study [40]. First, deletion-plasmid was constructed. Fragment A, located upstream of the gene *gad*R, and fragment B, located downstream of the gene *gad*R, were amplified from the genomic DNA of *L. brevis* using the primer pairs F-A-up-BamHI/R-A-up and F-B-down/R-B-down-*Hin*dIII, respectively (Table 2). To increase the recombination efficiency and not affect another gene expression beside the *gad*R, the length of fragment A or fragment B was designed to be 1000 bp separately containing 21 bp in the front or back regions of *gad*R. The construction of recombination fragment AB was performed by one-step fusion PCR. Fragment AB was digested by the enzymes *Bam*HI and *Hin*dIII and then cloned into pGID023 plasmid [38]. The ligation mixture was transferred into *E. coli* Top10, and deletion-plasmid pGID023-AB (9.9 kb) was obtained. Second, plasmid pGID023-AB was electroporated into *L. brevis*. A single colony was inoculated in 4 mL of MRS liquid medium supplemented with 1 μg/mL of erythromycin followed by incubating for 24 h at 37 °C. Then 10 μL of cell suspension was inoculated in same and fresh medium for cell passage. Campbell-type integration of pGID023-AB into the *L. brevis* chromosome via the fragment A or fragment B region resulted in tandem of plasmid and genome. The first integration was achieved by continuous cell passages (8 times) in MRS medium containing 1 μg/mL erythromycin. Secondary excision by intrachromosomal recombination via the fragment B or fragment A region resulted in a complete deletion of the *gad*R [41]. The second excision was achieved by cell continuous passage (10 times) in MRS medium without erythromycin. The primer pair F-367-0076/R-367-0078 was used to verify single crossover recombination, and the primer pair F-GadR/R-GadR was used to verify double crossover recombination (Table 2). The verified *gad*R deletion mutant was designated as Δ*gad*R.

### Screening of GABA-producing strains

The acidic fermented grains were collected from the traditional Chinese light aroma-type liquor production. Five grams of fermented grains were suspended in 50 mL 0.9% NaCl solution and then incubated for 1 h at 200 rpm, 37 °C. Dilutions were made by 0.9% NaCl solution, aliquots (100 μL) of 10^−1^ to 10^−5^ dilutions were spread on MRS agar plates containing 1% (W/V) CaCO_3_ [42] and 10 g/L of MSG followed by incubating for 48 h at 37 °C in an anaerobic incubator. Single colonies showed transparent halos on agar plates were inoculated into MRS liquid medium containing 10 g/L MSG followed by incubating for 48 h at 37 °C in an anaerobic incubator.

The 16S rRNA gene of the screened GABA-producing bacteria was amplified by PCR using the primers pair F27/R1492 according to the reported approach [43]. The amplified DNA fragments were sequenced by the Sangon Biotech Co. Ltd. (Shanghai, China) and then subjected to BLAST (https://blast.ncbi.nlm.nih.gov/Blast.cgi) against 16S rRNA sequences database to identify the species of screened strains.

### Fermentation

For small-scale culture, a single colony of *L. brevis* was inoculated into GYP medium in a 50-mL flask with 10 mL of working volume and was incubated for 24 h at 37 °C. The cell suspension was inoculated into 100 mL of GYP medium in a 250-mL flask and then cultivated for 15 h at 37 °C, 200 rpm as the seed culture. Twenty milliliters of seed culture were inoculated into 200 mL GYP medium containing 50 g/L of MSG in a 500-mL flask and then was incubated for 48 h at 37 °C, 200 rpm. Samples were collected at appropriate time points.

A 3-L fermenter (Eppendorf BioFlo/Celligen 115; Hamburg, Germany) was used in fed-batch fermentation and mixed-feed fermentation. One hundred milliliters (10%, V/V) seed culture were inoculated into GYP medium with 1 L’s working volume supplemented with 30 g/L of glucose as carbon source. MSG was added at a concentration of 74.8 g/L and then the pH was adjusted to 5.0 by addition of H_2_SO_4_. The temperature was maintained at 37 °C and the pH was maintained at 5.0 by automatic addition of 5 M H_2_SO_4_. The agitation speed was set to 200 rpm without gas sparging. Solutions of MSG (74.8 g/100 mL) were separately added into fermenter at 6 h, 12 h, 18 h and 24 h. For mixed-feed fermentation, solution of glucose (300 g/L) was fed at 5 mL/h using a peristaltic pump (LongerPump, Baoding, China) operating from 12 to 36 h.

### Analytical methods

Bacterial cell growth was monitored by measuring the optical density at 600 nm (OD_600_) with a spectrophotometer (AOE instruments A380, Shanghai, China).

The concentration of GABA in the culture broth was analyzed by high performance liquid chromatography (HPLC) with the *o*-phthaldialdehyde derivatization method [44]. Cell-free supernatant was filtered through a 0.45 μm membrane filter (Millipore, USA). GABA was detected by using the pre-column derivatization of Agilent HPLC system (Agilent 1200, USA) equipped with Agilent Zorbax Eclipse AAA column (Agilent, USA) according to the manufacturer’s instructions. GABA concentration was calculated from the integrated peak area comparing with standard curve constructed using GABA standard (Sigma, Aldrich Co., St. Louis, MO, USA).

The concentration of glucose in the culture broth was analyzed by HPLC (Agilent 1200, USA) system equipped with Aminex HPX-87H column (300 × 7.8 mm; BioRad) [45]. Samples were eluted by 5 mM H_2_SO_4_ with a flow of 0.60 mL/min and detected by refractive index detector. The temperature of the column was maintained at 60 °C.

## Additional files

**Additional file 1: Figure S1.** Effect of *gad*R-deletion on *Lactobacillus brevis* ATCC 367. (a) Identification of *gad*R-deletion in *L. breivs* ATCC 367 (367). (b) Expression level of *gad*R in strain 367 and 367 *gad*R-deletion mutant (367Δ*gad*R).

**Additional file 2: Figure S2.** GABA-producing strains were isolated from acidic fermented grains of Chinese liquor production. *L. h*, *Lactobacillus* (*L.*) *hilgardii*; *L. p*, *L. plantarum*; *L. pb*, *L. parabuchnery*; *L. b, L. brevis*.

**Additional file 3: Table S1.** List of representative GABA-producing lactic acid bacteria strains.

**Additional file 4: Figure S3.** Disruption of *gadR* eliminates GABA production in *L. brevis* D17 isolated from acidic habit.

**Additional file 5: Figure S4.** (a) Phylogenetic tree based on 16S rRNA gene sequence analysis. Bootstrap values were calculated from 1000 replications and these values were shown at branch point. (b) Gene loci encoding the proteins GadR, GadC and GadB in the genomes of lactic acid bacteria from NCBI. Numbers indicated protein identify.

## Data Availability

All data generated or analyzed during this study are included in this published article and in its additional files.

## Abbreviations

*L. brevis*: *Lactobacillus brevis*
*Lc. lactis*: *Lactococcus lactis*
*S. thermophilus*: *Streptococcus thermophilus*
GABA: γ-aminobutyric acid
LAB: lactic acid bacteria
GAD: glutamate decarboxylase
GRAS: generally-recognized-as-safe
PMF: proton motive force
*gad*CB: *gad*C-*gad*B operon
MSG: monosodium glutamate
NCBI: National Center for Biotechnology Information
qRT-PCR: quantitative reverse transcription PCR
HPLC: high performance liquid chromatography
