# Supplementary material for: Deciphering the crucial roles of transcriptional regulator GadR on gamma-aminobutyric acid production and acid resistance in Lactobacillus brevis
Source: Microb Cell Fact. 2019 Jun 13;18:108. doi: 10.1186/s12934-019-1157-2 (PMC6567505; doi:10.1186/s12934-019-1157-2)

**Title: Deciphering the crucial roles of transcriptional regulator GadR on gamma-aminobutyric acid production and acid resistance in *Lactobacillus brevis***

**Authors:** Luchan Gong^1, a^, Cong Ren^1, b^, Yan Xu^1, c*^

**Institutional address:**

1. Key Laboratory of Industrial Biotechnology of Ministry of Education, State Key Laboratory of Food Science and Technology, School of Biotechnology, Jiangnan University, Wuxi 214122, China

**Email addresses:**

a. [7140201001@vip.jiangnan.edu.cn](mailto:7140201001@vip.jiangnan.edu.cn)

b. [congren@jiangnan.edu.cn](mailto:congren@jiangnan.edu.cn)

c. [yxu@jiangnan.edu.cn](mailto:yxu@jiangnan.edu.cn)

***Corresponding Author:** Yan Xu

**Additional file 1**

**Figure S1.** Effect of *gad*R-deletion on *Lactobacillus brevis* ATCC 367. (a) Identification of *gad*R-deletion in *L. breivs* ATCC 367 (367). (b) Expression level of *gad*R in the strain 367 and 367 *gad*R-deletion mutant (367Δ*gad*R).


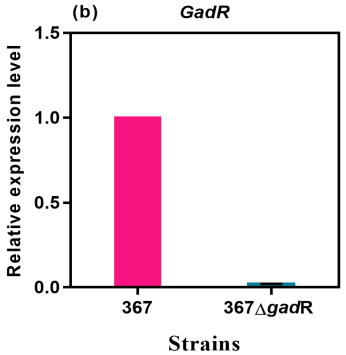

Supplement: Supplementary file 1 — Additional file 1: Figure S1. Effect of gadR-deletion on Lactobacillus brevis ATCC 367. (a) Identification of gadR-deletion in L. breivs ATCC 367 (367). (b) Expression level of gadR in strain 367 and 367 gadR-deletion mutant (367ΔgadR). [file 12934_2019_1157_MOESM1_ESM.docx]
