# Supplementary material for: Deciphering the crucial roles of transcriptional regulator GadR on gamma-aminobutyric acid production and acid resistance in Lactobacillus brevis
Source: Microb Cell Fact. 2019 Jun 13;18:108. doi: 10.1186/s12934-019-1157-2 (PMC6567505; doi:10.1186/s12934-019-1157-2)

**Additional file 2**

**Figure S2.** GABA-producing strains were isolated from acidic fermented grains of Chinese liquor production. *L. h*, *Lactobacillus(L.) hilgardii*; *L. p*, *L. plantarum*; *L. pb*, *L. parabuchnery*; *L. b, L. brevis.*


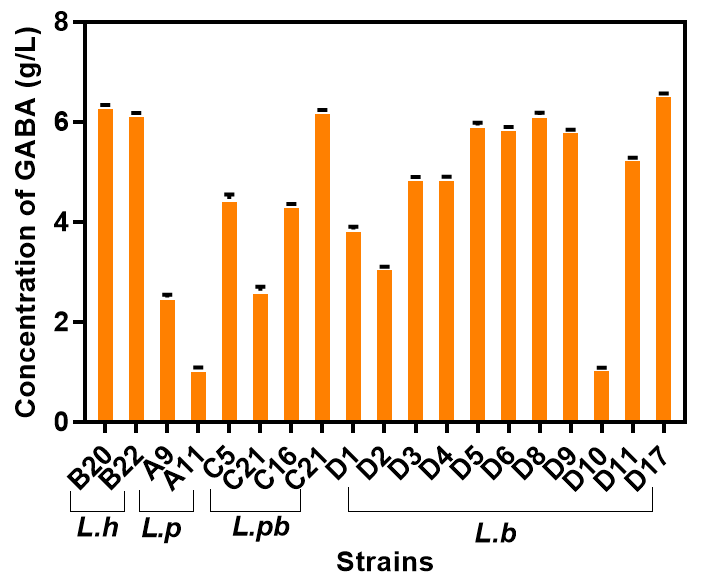

Supplement: Supplementary file 2 — Additional file 2: Figure S2. GABA-producing strains were isolated from acidic fermented grains of Chinese liquor production. L. h, Lactobacillus (L.) hilgardii; L. p, L. plantarum; L. pb, L. parabuchnery; L. b, L. brevis. [file 12934_2019_1157_MOESM2_ESM.docx]
