# Supplementary material for: Deciphering the crucial roles of transcriptional regulator GadR on gamma-aminobutyric acid production and acid resistance in Lactobacillus brevis
Source: Microb Cell Fact. 2019 Jun 13;18:108. doi: 10.1186/s12934-019-1157-2 (PMC6567505; doi:10.1186/s12934-019-1157-2)
Supplement: Supplementary file 3 — Additional file 3: Table S1. List of representative GABA-producing lactic acid bacteria strains. [file 12934_2019_1157_MOESM3_ESM.docx]

Additional file 3

**Table S1.** List of representative GABA-producing lactic acid bacteria strains.

| Strain | Origin | GABA | | Reference |
| --- | --- | --- | --- | --- |
|  |  | Titer  (g/L) | Productivity  (g/L/h) |  |
| *Lactococcus lactis* ssp. *lactis* 01-7 | cheese | 0.027 | 0.0000803 | [1] |
| *Lc.* *lactis* ssp *lactis* CECT 8184 | cheese | 0.1 | 0.0042 | [2] |
| *Lb. brevis* | cheese | 0.13 | 0.0027 | [3] |
| *Lb. plantarum* NDC75017 | yogurt | 3.14 | 0.065 | [4] |
| *Lb. rhamnosus* GG | adzuki bean milk | 1.12 | 0.03 | [5] |
| *Lb. brevis* NCL912 | *paocai* | 15.4 | 0.32 | [6] |
| *Lb. brevis* NCL912 | *paocai* | 103 | 2.14 | [7] |
| *Lb. brevis* TCCC13007 | pickled vegetables | 61.0 | 0.92 | [8] |
| *Lb. brevis* GABA 100 | kimchi | 25.4 | 0.071 | [9] |
| *Lb. brevis* BJ20 | kimchi | 2.47 | 0.02 | [10] |
| *Lc. lactis* B | kimchi | 6.41 | 0.044 | [11] |
| *Lb. buchneri* MS | kimchi | 25.85 | 0.72 | [12] |
| *Lb. brevis* AML15 | kimchi | 10.7 | 0.22 | [13] |
| *Lb. brevis* BFC-110 | kimchi | 26.7 | 0.37 | [14] |
| *Lb. brevis* | kimchi | 44.4 | 0.62 | [15] |
| *Lb. brevis* NPS-QW-145 | kimchi | 25.83 | 0.36 | [16] |
| *Enterococcus avium* 9184 | fermented scallop | 3.71 | 0.04 | [17] |
| *Lb. brevis* RK03 | saltwater fish | 62.52 | 0.71 | [18] |
| *Lb. futsaii* CS3 | fermented shrimp | 13.1 | 0.18 | [19] |
| *Enterococcus avium* M5 | fermented seafood | 18.47 | 0.38 | [20] |
| *Lb. paracasei* NFRI 7415 | fermented fish | 31.11 | 0.19 | [21] |
| *Lb. brevis* CRL 1942 | sourdough | 26.27 | 0.55 | [22] |
| *Lb. plantarum* Taj-Apis362 | honeybees | 0.736 | 0.012 | [23] |
| *Pediococcus pentosaceus* HN8 | fermented foods | 9.06 | 0.38 | [24] |
| *Lb. brevis* ATCC367 | silage | 9.65 | 0.20 | This study |
| *Lb. brevis* D17 | fermented grains | 177.8 | 4.93 | This study |

**References**

1. Nomura M, Kimoto H, Someya Y, Furukawa S, Suzukl I. Production of γ-aminobutyric acid by cheese starters duringcheese ripening. J Dairy Sci. 1998;81:1486–1491.

2. Diana M, Tres A, Quílez J, Llombart M, Rafecas M. Spanish cheese screening and selection of lactic acid bacteria with high gamma-aminobutyric acid production. LWT-Food Sci Technol. 2014;56:351-355.

3. Carafa I, Nardin T, Larcher R, Viola R, Tuohy K, Franciosi E. Identification and characterization of wild lactobacilli and pediococci from spontaneously fermented mountain cheese. Food Microbiol. 2015;48:123-132.

4. Shan Y, Man CX, Han X, Li L, Guo Y, Deng Y, Li T, Zhang LW, Jiang YJ. Evaluation of improved gamma-aminobutyric acid production in yogurt using *Lactobacillus plantarum* NDC75017. J Dairy Sci. 2015;98:2138-2149.

5. Song HY, Yu RC. Optimization of culture conditions for gamma-aminobutyric acid production in fermented adzuki bean milk. J Food Drug Anal. 2018;26:74-81.

6. Li HX, Gao DD, Cao YS, Xu HY. A high γ-aminobutyric acid-producing *Lactobacillus brevis* isolated from Chinese traditional paocai. Ann Microbiol. 2008;58:649-653.

7. Li HX, Qiu T, Huang GD, Cao YS. Production of gamma-aminobutyric acid by *Lactobacillus brevis* NCL912 using fed-batch fermentation. Microb Cell Fact. 2010;9:85-92.

8. Zhang Y, Song L, Gao Q, Yu SM, Li L, Gao NF. The two-step biotransformation of monosodium glutamate to GABA by *Lactobacillus brevis* growing and resting cells. Appl Microbiol Biotechnol. 2012;94:1619-1627.

9. Kim JY, Lee MY, Ji GE, Lee YS, Hwang KT. Production of gamma-aminobutyric acid in black raspberry juice during fermentation by *Lactobacillus brevis* GABA100. Int J Food Microbiol. 2009;130:12-16.

10. Lee BJ, Kim JS, Kang YM, Lim JH, Kim YM, Lee MS, Jeong MH, Ahn CB, Je JY. Antioxidant activity and γ-aminobutyric acid (GABA) content in sea tangle fermented by *Lactobacillus brevis* BJ20 isolated from traditional fermented foods. Food Chem. 2010;122:271-276.

11. Lu X, Chen Z, Gu Z, Han Y. Isolation of γ-aminobutyric acid-producing bacteria and optimization of fermentative medium. Biochem Eng J. 2008;41:48-52.

12. Cho YR, Chang JY, Chang HC. Production of gamma-aminobutyric acid (GABA) by *Lactobacillus buchneri* isolated from kimchi and its neuroprotective effect on neuronal cells. J Microbiol Biotechnol. 2007;27:104-109.

13. Shin JW, Kim DG, Lee YW, LEE HS, Shin KS, Choi CS, Kwon GS. Isolation and characterization of *Lactobacillus brevis* AML15 producing γ-aminobutyric acid. J Life Sci. 2007;17:970-975.

14. Kim DS: Study on the fermentation in lactic acid bacteria for the production of γ-aminobutyric acid**.** Hannam Univ., Daejeon; 2009.

15. Binh TTT, Ju WT, Jung WJ, Park RD. Optimization of gamma-amino butyric acid production in a newly isolated *Lactobacillus brevis*. Biotechnol Lett. 2014;36:93-98.

16. Wu QL, Shah NP. Gas release-based prescreening combined with reversed-phase HPLC quantitation for efficient selection of high-gamma-aminobutyric acid (GABA)-producing lactic acid bacteria. J Dairy Sci. 2015;98:790-797.

17. Yang HY, Xing RE, Hu LF, Liu S, Li PC. Accumulation of gamma-aminobutyric acid by *Enterococcus avium* 9184 in scallop solution in a two-stage fermentation strategy. Microb Biotechnol. 2016;9:478-485.

18. Wu CH, Hsueh YH, Kuo JM, Liu SJ. Characterization of a potential probiotic *Lactobacillus brevis* RK03 and efficient production of gamma-aminobutyric acid in batch fermentation. Int J Mol Sci. 2018;19:1-16.

19. Sanchart C, Rattanaporn O, Haltrich D, Phukpattaranont P, Maneerat S. *Lactobacillus futsaii* CS3, a new GABA-producing strain isolated from Thai fermented shrimp (kung-som). Indian J Microbiol. 2017;57:211-217.

20. Lee KW, Shim JM, Yao Z, Kim JA, Kim HJ, Kim JH. Characterization of a glutamate decarboxylase (GAD) from *Enterococcus avium* M5 isolated from jeotgal, a Korean fermented seafood. J Microbiol Biotechnol. 2017;27:1216-1222.

21. Komatsuzaki N, Shima J, Kawamoto S, Momose H, Kimura T. Production of γ-aminobutyric acid (GABA) by *Lactobacillus paracasei* isolated from traditional fermented foods. Food Microbiol. 2005;22:497-504.

22. Villegas JM, Brown L, Savoy de Giori G, Hebert EM. Optimization of batch culture conditions for GABA production by *Lactobacillus brevis* CRL 1942, isolated from quinoa sourdough. LWT-Food Sci Technol 2016;67:22-26.

23. Tajabadi N, Ebrahimpour A, Baradaran A, Rahim RA, Mahyudin NA, Manap MYA, Bakar FA, Saari N. Optimization of gamma-aminobutyric acid production by *Lactobacillus plantarum* Taj-Apis362 from honeybees. Molecules. 2015;20:6654-6669.

24. Ratanaburee A, Kantachote D, Charernjiratrakul W, Sukhoom A. Selection of γ-aminobutyric acid-producing lactic acid bacteria and their potential as probiotics for use as starter cultures in Thai fermented sausages (Nham). Int J Food Sci Technol. 2013;48:1371-1382.
