# Supplementary material for: Deciphering the crucial roles of transcriptional regulator GadR on gamma-aminobutyric acid production and acid resistance in Lactobacillus brevis
Source: Microb Cell Fact. 2019 Jun 13;18:108. doi: 10.1186/s12934-019-1157-2 (PMC6567505; doi:10.1186/s12934-019-1157-2)

**Additional file 4**

**Figure S3.** Disruption of *gadR* eliminates GABA production in *L. brevis* D17 isolated from acidic habit.


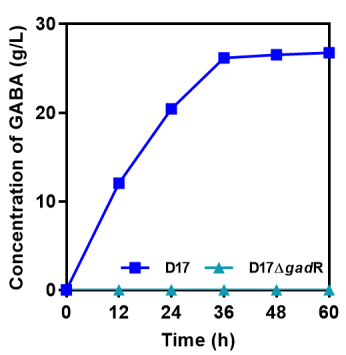

Supplement: Supplementary file 4 — Additional file 4: Figure S3. Disruption of gadR eliminates GABA production in L. brevis D17 isolated from acidic habit. [file 12934_2019_1157_MOESM4_ESM.docx]
