# Supplementary material for: Deciphering the crucial roles of transcriptional regulator GadR on gamma-aminobutyric acid production and acid resistance in Lactobacillus brevis
Source: Microb Cell Fact. 2019 Jun 13;18:108. doi: 10.1186/s12934-019-1157-2 (PMC6567505; doi:10.1186/s12934-019-1157-2)

Additional file 5

**Figure S4.** (a) Phylogenetic tree based on 16S rRNA gene sequence analysis. Bootstrap values were calculated from 1000 replications and these values were shown at branch point. (b) Gene loci encoding the proteins GadR, GadC and GadB in the genomes of lactic acid bacteria from NCBI. Numbers indicated protein identify.


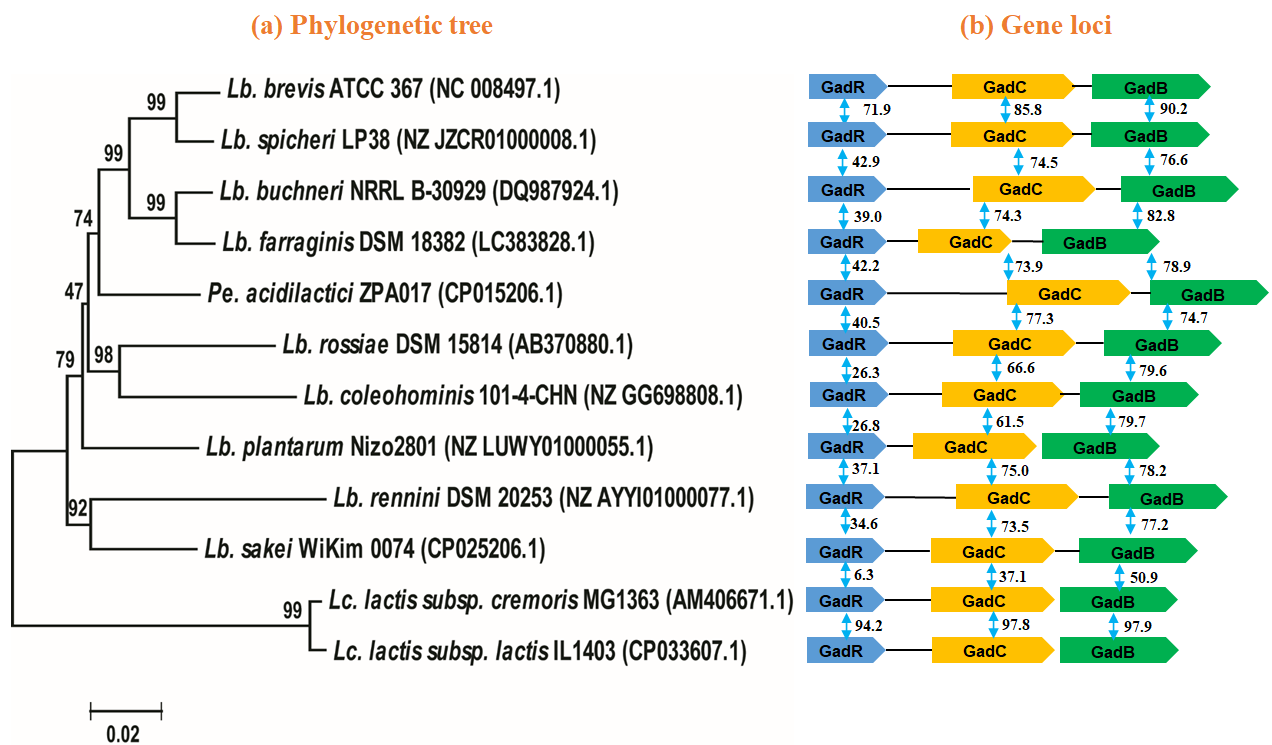

Supplement: Supplementary file 5 — Additional file 5: Figure S4. (a) Phylogenetic tree based on 16S rRNA gene sequence analysis. Bootstrap values were calculated from 1000 replications and these values were shown at branch point. (b) Gene loci encoding the proteins GadR, GadC and GadB in the genomes of lactic acid bacteria from NCBI. Numbers indicated protein identify. [file 12934_2019_1157_MOESM5_ESM.docx]
